# An arbuscular mycorrhizal fungus alters soil water retention and hydraulic conductivity in a soil texture specific way

**DOI:** 10.1007/s00572-023-01106-8

**Published:** 2023-03-28

**Authors:** Richard Pauwels, Jan Graefe, Michael Bitterlich

**Affiliations:** 1 Leibniz Institute of Vegetable and Ornamental Crops e.V. (IGZ), Grossbeeren, Germany; 2 Division Urban Plant Ecophysiology, Thaer-Institute, Humboldt-Universität zu Berlin, Berlin, Germany

**Keywords:** Arbuscular mycorrhiza, Hydraulic conductivity, Maize, *Rhizophagus irregularis*, Soil texture, Water retention

## Abstract

Arbuscular mycorrhizal fungi (AMF) alter plant water relations and contribute to soil structure. Although soil hydraulic properties depend on soil structure and may limit plant water uptake, little is known about how AMF influence soil water retention (the relation between the soil water content and soil water potential) and hydraulic conductivity in different soils. Instead, these soil hydraulic properties often are considered to be independent of AMF presence in experiments. We asked if this assumption holds true for both sand and loam. We grew maize plants either inoculated with *Rhizophagus irregularis* or with autoclaved inoculum in pots filled with quartz sand or loam soil until extraradical spread of the fungus throughout the pots was achieved. Each pot contained a hyphal compartment made of a soil sampling core (250 cm^3^) covered with a 20-µm nylon mesh to encourage fungus ingrowth but to exclude root ingrowth. We measured soil water retention and unsaturated hydraulic conductivity in these undisturbed root-free soil volumes. We observed that in loam harboring the mycorrhizal fungus, the soil water retention decreased, while in sand, it increased without detectable changes in the soil bulk density. The effects of the fungus on the soil water potential were strongest at low soil water contents in both soils. As a consequence of the altered water potentials in soils with the mycorrhizal fungus, soil hydraulic conductivity increased in loam but decreased in sand after fungus ingrowth. We conclude that in our study, the mycorrhizal fungus acted as a soil conditioner even distant from roots, which encouraged drainage in loams prone to sogginess but enhanced water storage in sands prone to quick desiccation. We recommend considering soil hydraulic properties as being dynamic in future studies on water relations of mycorrhizal plants.

**Supplementary Information:**

The online version contains supplementary material available at 10.1007/s00572-023-01106-8.

## Introduction

Arbuscular mycorrhizal fungi (AMF) influence plant water relations. There is abundant evidence for this from hundreds of studies over past decades. This has led to the interpretation that AMF may confer drought tolerance to plants. But “drought tolerance” is a term difficult to define. Hence, the mycorrhizal symbiosis probably is better interpreted as an emergent property for improved scavenging of water from (dry) soils than as a condition that confers obvious physiological drought tolerance such as succulence or Crassulacean acid metabolism. In light of this, extensive reviews have shown that, more often than not, mycorrhizal plants adjusted their drought physiology later during soil drying or in comparatively drier soils than their non-mycorrhizal counterparts (Augé 2001; Augé et al. 2015). In search for the underlying mechanisms, studies have revealed a complex reorganization of the mycorrhizal plant water stress response, involving sustained stomatal opening (Augé et al. 2015), higher plant water potentials (Abdalla and Ahmed 2021; Porcel and Ruiz-Lozano 2004), differential expression and activation of root aquaporins (Sharma et al. 2021), altered osmolyte contents (Bárzana et al. 2015; Begum et al. 2020), and enhanced root hydraulic conductivities in dry soils (Aroca et al. 2007; Sánchez-Romera et al. 2016). These observations may indicate that more water can reach a mycorrhizal root system than a non-mycorrhizal one growing in dry soils. In fact, trace labeling studies showed that isotopically labeled water molecules only accessible to AMF hyphae can pass through host plants (Püschel et al. 2020), while the movement along hyphae mainly is passive, outside the hyphal cell membrane (Kakouridis et al. 2022). Still, the estimated water quantities delivered from a root-free soil volume containing AMF hyphae (hyphosphere) have varied substantially: from physiologically minor relevance (George et al. 1992; Khalvati et al. 2005; Püschel et al. 2020) to more than 20% of transpired water (Faber et al. 1991; Kakouridis et al. 2022; Ruth et al. 2011). This high variation in outcomes was irrespective of whether the contribution of AMF to plant water uptake was quantified directly by fates of isotopes or indirectly by monitoring soil water depletion rates.

Such strong context dependency can have various reasons. Among them are the variability of AMF abundances and the soil types used across studies and whether additional water from a hyphosphere is actually needed to compensate a shortage in water supply to the transpiration stream. Whether the soil-to-root water flow in dry soils limits plant water uptake or not directly depends on the size of the root system and the soil hydraulic properties (Carminati et al. 2020; Graefe et al. 2019). Besides distinct root growth patterns of mycorrhizal plants, soil intrinsic hydraulic properties, i.e., soil water retention and soil hydraulic conductivity, may change upon AMF ingrowth into the soil pore space. Indeed, there are indications of such changes (Augé et al. 2001; Bitterlich et al. 2018a; Pauwels et al. 2020).

Soil water retention (the relation between the soil water content and the soil water potential) and hydraulic conductivity (the inverse of the water flow resistance in soils) set limits to plant water extraction from soils. Both soil hydraulic traits depend on soil texture, soil structure, and the wetting properties of the solid soil phase. Soil texture, i.e., the proportional mix of large sand (2000–50 µm), intermediate silt (50–2 µm), and small clay (< 2 µm) particles in soils (according to the USDA), constrains soil water retention and hydraulic conductivity as particle sizes give rise to soil pore sizes. A soil dominated by large sand particles has a low water retention capacity but a high hydraulic conductivity because the fraction of quickly drainable large pores dominates, and smaller pores that retain water against gravity are lacking. Vice versa is true for clayey, fine-textured soils. A balanced mix of the three particle size classes, i.e., a loam soil, consequently shows intermediate water retention and hydraulic conductivity. Within a certain soil texture, water retention and hydraulic conductivity further differ with soil structure, i.e., the secondary three-dimensional particle arrangement. Soil structure involves the formation of organo-mineral soil aggregates (Tisdall and Oades 1982), a process that is well known for being dynamic in time and supported by AMF (Rillig and Mummey 2006). The internal pore space of large aggregates is considered to be a main habitat for soil fungi and AMF increase the stability of these aggregates (Miller and Jastrow 2000; Rillig and Mummey 2006). How extensively soils contain aggregates upon biotic influences such as AMF ingrowth in turn depends on soil texture (Nimmo 2004a). The higher the amount of low weight (clay) particles with charged surfaces in a soil, the more susceptible the soil is to aggregation because light particles are more easily movable and more tightly bound into organo-mineral complexes than are heavy particles (Nimmo 2004a). As a consequence, aggregation of soil particles from a single-grain texture into organo-mineral complexes influences soil water retention and hydraulic conductivity because the corresponding pore space changes in size and geometry (Guber et al. 2003, 2004; Nimmo 2004b). These soil physical principles suggest that soil water retention and hydraulic conductivity are likely to respond to AMF ingrowth and that their response to AMF presence is specific to soil texture. Despite this, it is not well known how AMF affect soil water retention and hydraulic conductivity quantitatively in soils of different textures (Querejeta 2017).

Because soil hydraulic properties are likely to be influenced when AMF grow into pores that previously were devoid of them, the question is not if AMF affect soil hydraulic properties but how strongly they do so. Hitherto, the answer to this question has remained elusive. Soil water retention by soils containing AMF has been investigated in only a few instances, and soil hydraulic conductivity, to our best knowledge, just two times overall and only in artificial potting mixes (Bitterlich et al. 2018a, b). A study that compares AMF influences on water retention and hydraulic conductivity in different soils is lacking.

To this date, soil water retention and hydraulic conductivity are treated almost exclusively as a constant when plant water relations in the mycorrhizal state are investigated. Usually, an implicit assumption is made. That is that irrigation to equal soil water potentials or water contents in non-mycorrhizal and mycorrhizal pots induces equal soil water stress. This, however, may not be the case as soon as AMF influence water retention and/or hydraulic conductivity of the soil. To investigate if this assumption holds true, we ask in this study whether soil water retention and hydraulic conductivity are changed by the presence of hyphae of an arbuscular mycorrhizal fungus, and we discuss whether this potentially influences the water stress experienced by plants. In particular, we assumed that the influences of *Rhizophagus irregularis* on soil water retention and hydraulic conductivity depend on soil texture. We hypothesized that the fungus would reduce soil water retention and increase hydraulic conductivity in a flexible, fine-textured loam but that it would increase water retention and reduce hydraulic conductivity in a rigid, coarse-textured sand. We chose loam and sand to contrast soil texture because they are either susceptible or widely resistant to aggregation, respectively, but both can be irrigated effectively in pots (as opposed to a heavy clay).

## Material and methods

We aimed to quantify the AMF influence on soil hydraulic properties of the two different soils in the absence of roots, i.e., in the hyphosphere. For this, we implanted hyphal compartments in pots with maize. We measured water retention and hydraulic conductivity in the hyphal compartment soil. As a proxy for plant access to hyphosphere water, we tested how the root-excluding nylon mesh we used for construction of the hyphal compartments affected hydraulic conductivity between the two soil volumes that it separated. In order to investigate how the mycorrhizal fungus modulated soil hydraulic properties in a texture specific way, we chose two soils that are similarly poor in organic matter. Thus, we assured that newly introduced organic matter of mycorrhizal fungus origin was deposited in divergent mineral matrices but added similar proportions of soil organic matter. Because of the different physico-chemical properties of loam and sand, we applied soil specific fertilization and irrigation in order to generate symbiotic plants that sustained similar abundances of the mycorrhizal fungus in the soil. Afterwards, we analyzed plant biomass and nutrition in order to verify that the desired relative plant P limitation (under which AMF usually thrive) was achieved to similar extents in both soils. To further minimize differences in final mycorrhizal fungus soil abundances, we anticipated that different growth durations would be required to achieve similar concentrations of mycorrhizal fungus in both soils. To adjust for this, we implanted removable biopsy pockets filled with dead trap roots into the soils at the farthest distance from plants. Those quick and easy-to-screen trap roots were used to determine the harvest dates of plants. As soon as colonization of the distant trap roots with mycorrhizal fungus storage structures was observed, plants were harvested.

### Experimental design and preparation of pots

We carried out two sequential inoculation experiments in a glass house with *Zea mays* cv. “FARMFIRE” (Austria, Control-Nr. A6R5068), one experiment with a loam (as classified by the USDA, as a silty loamy sand following the German classification KA5) and the other with a fine quartz sand from a surface mine (51.20°N, 13.8°E). The loam is a Luvisol obtained from a C-horizon in Weihenstephan (48.42°N, 11.8°E) with 35% sand, 50% silt, 15% clay, 0.3% organic matter (w/w), and a pH of 7.2 (CaCl_2_). It contained 89 mg kg^−1^ CaCl_2_-extractable Mg, 27.6 mg kg^−1^ and 39 mg kg^−1^ acetate-lactate-extractable P and K, respectively. The loam also contained CaCl_2_/DTPA-extractable 0.6 mg kg^−1^ Cu, 19 mg kg^−1^ Mn, and 0.3 mg kg^−1^ Zn. The washed quartz sand had a grain size of 0.2–1 mm and non-detectable plant mineral nutrients. The soils were dry heated (85 °C, 48 h) to eliminate preexisting fungal propagules and homogenized (2 mm sieved). For each soil, eight 3 l pots were filled with 3 kg of soil and compacted to final bulk densities of 1.2 g cm^−3^ in pots with loam and of 1.55 g cm^−3^ in pots with quartz sand. We used non-perforated pots to prevent drainage and leaching of nutrients during the experiment. The pots of the loam were amended with: N, 200; K, 232; Mg, 40; Ca, 153, P, 3 [mg kg^−1^] in aqueous solution, thoroughly mixed with the soil before pot filling. For the quartz sand, a nutrient solution for hydroponics was prepared (De Kreij et al. 1997) with 10% of the standard [P] and a pH of 6.2. The solution was mixed with the sand in quantities that provided the same macronutrient contents as added to the loam, except for P. After the 6th week of growth, we added an additional 25 mg of P (KH_2_PO_4_) to each pot filled with loam and 1.5 mg kg^−1^ of P to each pot filled with sand. In total, the pots with the strongly P fixing loam were provided 600 mg N and 117 mg P for growth, while the pots with sand accommodated 600 mg N and 13.5 mg P. These conditions were aimed to provide relative P limitation to plants and good AMF development regardless of the P fixing capacity of the soil.

To set up the mycorrhizal treatment (AM), 4 of 8 pots for each soil were inoculated with 5% (v/v) of a sand-based inoculum containing colonized root pieces and spores of *Rhizophagus irregularis* QS69 (Inoq GmbH, Schnega, Germany). For the non-mycorrhizal treatment (NM), the other half of the 8 pots received an equal proportion of autoclaved inoculum (121 °C, 30 min) and a previously acquired bacterial filtrate of the inoculum to encourage a consistent microflora in non-mycorrhizal pots. For every NM pot, the bacterial filtrate was produced by filtering 200 ml of deionized water through a Whatman filter (particle retention 4–7 μm; GE Healthcare Europe GmbH, Freiburg, Germany) containing approx. 100 ml of live inoculum. The same amount of deionized water was added to each of the AM pots.

### Hyphal compartments

In order to study the influences of mycorrhizal fungus ingrowth on soil hydraulic properties, each pot contained a compartment that allowed undisturbed harvesting of root-free but mycorrhizal fungus-containing soil volumes (hyphal compartments). The compartments were constructed with 250 ml steel cylinders (*h* = 5 cm, *r* = 4 cm) that are commonly used for soil sampling in the field (Eijkelkamp, the Netherlands). The cores with 50 cm^2^ openings at top and bottom were filled with the pot soil and compressed to the same bulk density as in the pots. The core openings then were covered with a 20-µm nylon mesh (Sefar AG, Switzerland) to prevent root ingrowth. The compartment soil was not inoculated. Hence, all fungal structures found inside the compartments would originate from mycorrhizal fungus proliferation, and the soil texture in the hyphal compartments was not compromised by inoculum addition. The meshes also served as a safety net for undisturbed harvesting of the sampling cores. The compartments were introduced into the center of the pots with the core openings aligned upright in the vertical direction. This guaranteed that AMF hyphae could populate soils inside the hyphal compartments by spreading laterally from downward growing roots.

### Trap root compartments

The pots also contained trap root compartments to further verify fungus spread throughout the pots as difficulties in extraction of hyphae from soils of different textures were anticipated. We also considered that mycorrhizal fungus growth in the two soils would require different durations to comprehensively colonize the pot soil. To adjust for that, we introduced easy-to-remove trap root compartments that allowed us to decide on the harvest date. For this, we followed the procedure of Müller et al. (2017). For the production of trap roots, maize plants were cultivated in open pots on quartz sand to obtain non-colonized roots. The roots were washed, formed into a flat root mat, and air dried at 27 °C for 24 h. The root mats were cut into rectangles that fit biopsy pockets. The biopsy pockets were covered with the same nylon mesh as used to for the hyphal compartments and were introduced into the pots (vertically covering 5 to 10 cm depth from the soil surface and approximately 2 cm distant from the pot rim) approximately 2 weeks before harvesting the experiments. Place holders guaranteed that introduction and removal of the biopsy pockets did not disturb the pot soil. The plants grown on both soils were harvested as soon as AMF storage organs could be observed in the trap roots. This was the case at the end of the 11th week after planting in loam and at the end of the 10th week in sand. Afterwards, we checked whether our visual inspection was backed up by quantitative colonization data (see below). Please refer to Fig. S1 for an illustration of the compartment placement in each pot.

### Plant cultivation

For each of the two experiments, 4 arbuscular mycorrhizal pots (AM) and 4 non-mycorrhizal pots (NM) were set up randomly in a glass house and grown for 11 weeks on loam and 10 weeks on sand (for climatic conditions, see Fig. S2). We placed five seeds in each pot, and after reaching the two-leaf state, we retained one seedling of similar sizes in each pot. The shoots of the other seedlings were clipped.

Each of the 8 pots per experiment was placed on a separate pan of a modular multiplex balance system connected to a data logger (Campbell, USA) that monitored evapotranspiration losses every 30 min. Beforehand, we determined the water holding capacity of the pots filled with 3 kg of the different soils by capillary saturation for 24 h and by drainage in open pots for 24 h under exclusion of evaporation. We decided to grow plants at 50% of the water holding capacity, which amounted to 178 ml kg^−1^ dry soil for loam and 81 ml kg^−1^ for sand. This resulted in target volumetric water contents of 20% in pots with loam and of 12% in pots with sand. The specific pot water losses were compensated at least every other day with deionized water.

### Nutrient analyses

For nitrogen (N) and phosphorus (P) mass fractions in leaf and root tissues, dry heated (60 °C, 48 h) plant material was ground to a fine powder (ZM 200, Retsch GmbH, Haan, Germany). Samples of 10 mg were analyzed with elemental analysis (EA) for determination of N. For P, subsamples of 250 mg were suspended in 5 ml of HNO_3_ (65%) and 3 ml of H_2_O_2_ (30%). After a pre-reaction of 20–30 min, samples were transferred to a microwave for digestion. P concentrations were then determined colorimetrically by flow injection analysis (FIA). Two technical replicates were analyzed for each biological replicate.

### Quantification of arbuscular mycorrhizal colonization of roots and soils

Plant roots were sampled (approx. 1 g of a representative sample) and stained following Vierheilig et al. (1998). For this, roots were cleared in KOH solution (10%) at 60 °C for 30 min. After discarding the KOH solution, root samples were rinsed with tap water. Then, roots were acidified with HCl (2 N) for 2 min at room temperature. After disposal of the HCl, roots were incubated in 5% ink-acid solution (ink: Pelikan blue, acid: vinegar) at 60 °C for 40 min. Finally, roots were rinsed with tap water again and stored in lactic acid until analysis.

For quantification of the root colonization frequency (F%), mycorrhizal intensity in the root system (M%), and arbuscule abundance in the root system (A%), 50 fragments (of 1 cm length) of stained plant roots were scored under a bright-field microscope according to Trouvelot et al. (1986).

To extract the mycorrhizal fungus from the hyphal compartments, the compartment soil (250 ml) was suspended in 5 l of deionized water and stirred for 3 min. After heavy soil particles settled, the suspension with the floating AMF structures was poured through a 25-µm sieve. The mycelia were rinsed several times and collected in an Eppendorf tube. After lyophilizing, the hyphae were suspended with a few drops of ink and lactic acid. The stained samples were blended at low speed for 40 s (Waring Blender 7009G, Waring, USA) with 200 ml tap water. Ninety milliliter of the suspension was removed, and the length of hyphae and the number of AMF spores were determined by a membrane filter method as described by Hanssen et al. (1974). The extraction of the mycorrhizal fungus from the hyphal compartment soil was done after hydraulic property analyses were completed but before the soil was oven dried (see next section).

The roots from the trap root compartments in the biopsy pockets were harvested and quantified to verify our initial visual inspection and to confirm that enough time had elapsed for sporulation. Twenty root pieces of 3 mm length with a diameter smaller than 150 µm and 20 pieces with a diameter larger than 150 µm were randomly selected from each sample. The root pieces were stained for 40 min at 60 °C in 5% ink-acid solution (Pelikan blue, vinegar). Afterwards, AMF storage organs (vesicles and spores) in the trap roots were counted under a compound microscope (Axiolab 5, Zeiss, Germany; magnification 200 ×) to quantify the frequency of colonized trap roots and the quantity of fungal storage organs per infected root piece.

### Soil hydraulic properties

The hyphal compartments were carefully extracted from the pots, and the mesh was removed under avoidance of mechanical disturbance of the inner core soil. Subsequently, the soil cores were water saturated by capillary rise in a water bath for 24 h. The cores then were subjected to a simplified evaporation method following Schindler et al. (2010a) under lab conditions. For this, the sampling cores were mounted to a HYPROP system (HYPROP-System, Meter Group AG, Munich, Germany) according to the manufacturer’s manual (Pertassek et al. 2015). Two tensiometers were introduced into the soil volume in a way that one tensiometer tip was located at 1.25 cm the other at 3.75 cm height of the soil volume in the core (core height was 5 cm). The device then continuously logged the soil water potential (Ψ, kPa) sensed by the two tensiometers every 10 min during ongoing evaporation in the lab. The water loss was measured gravimetrically two times a day. Thereby, we obtained the continuous relationship between the volumetric water content (Θ) and the soil water potential (as the geometric mean of Ψ from both tensiometers), i.e., the soil water retention curve. When air entered the tensiometers, the measurement cycle was terminated. This happened between −80 and −150 kPa in loam and between −50 and −110 kPa in sand. An additional measurement point at −880 kPa was introduced into the relationship as this is a material constant of the porous tensiometer ceramic. This extends the measurement range (Schindler et al. 2010b). The method also obtained the relation between the soil hydraulic conductivity (K) and the soil water potential by assuming that half of the upwards water flow for evaporation derives from the lower half of the soil volume. After this and the extraction of hyphae from the soil, the soil from the cores was dried for 24 h at 105 °C to determine the dry weight.

The data were used to fit the Peters-Durner-Iden variant of the unimodal and constrained van Genuchten model (Iden and Durner 2014; Peters 2013; Peters et al. 2015) coupled to the Mualem hydraulic conductivity algorithm (Mualem 1986) with the LABROS-SoilView software (version 5.0.5.0, Meter-Group, Munich, Germany). Several hydraulic models were tested, and we chose the model we used based on objective criteria (lowest AICc, lowest RMSE). The model variant extrapolates to the thermodynamically expected water potential at zero water content, which improved the fit to the data at the dry end. For theory and equations of the model, you can refer to Pertassek et al. (2015). For visual inspection of the goodness of fit, please refer to Fig. S3 (for loam) and Fig. S4 (for sand).

In order to obtain a surrogate for the conditions in the planted pots, we additionally tested whether the nylon mesh that covered the hyphal compartments would impede water exchange between soil volumes. For this, we used the method described above and measured water retention and hydraulic conductivity in soils that contained a nylon mesh at half the distance between the two tensiometers. Please see Fig. S5 for a diagram. The soil core was filled with soil to half of the core height, and the mesh was placed horizontally on top of the soil. The mesh had a hole that matched the tensiometer cross-section. A place holder of tensiometer size was introduced, and then the top part of the core was filled with soil. The place holder was removed, and the tensiometers were introduced, resulting in one tensiometer tip located 1.25 cm below the mesh and the other tensiometer tip 1.25 cm above the mesh (Fig. S5). Controls without a mesh were handled identically, except they lacked the mesh placement (*N* = 3 for loam, *N* = 4 for sand).

### Statistics

For comparison of means between AM and NM treatments, Students *t* tests were used. In cases of data with unequal variances, the data were log transformed to meet the criterion of variance homogeneity. The differences were considered significant at threshold *α* = 0.05. The soils were not compared statistically because the experiments were not concurrent.

## Results

### Plant and mycorrhizal fungus development

We achieved intense root colonization of maize by *R. irregularis* in both loam soil and quartz sand (Table 1). NM roots and soils were free of AMF structures. Nearly all sampled root pieces were colonized in both soils, and the arbuscule abundance in roots was approx. 31% in loam and 27% in sand when inoculated. The trap roots were introduced to estimate extraradical proliferation across the two soils. We found that large trap roots with a diameter higher than 150 µm were preferred by the mycorrhizal fungus over small dead trap roots. Large trap roots were more frequently colonized and contained more storage organs than small trap roots. Overall, mycorrhizal development in living plant roots and dead trap roots in loam and sand was similar in both soils at the date of assessment. The colonization rates of trap roots were low because we terminated the experiments as soon as we observed AMF colonization in them.

**Table 1:** Mycorrhizal colonization in 10 to 11-week old maize pot cultures inoculated with *R. irregularis*. The values show the mean (± se) of four biological replicates each for maize cultivated in loam soil and in quartz sand. The location informs about root colonization traits in host plant roots (roots), in dead trap root compartments (trap roots) as a proxy for mycorrhizal fungus spread in pots, and mycorrhizal fungus abundance in hyphal compartments (soil core) that excluded root ingrowth (n.d., not determined because the hyphal dry matter in loam could not be accurately analyzed because of the attachment of clay minerals to hyphae)

Location	Variable	Loam	Sand
Roots	Colonization frequency in root system [F%]	93.25 ± 2.21	84.12 ± 2.62
	Mycorrhizal intensity in root system [M%]	52.89 ± 3.82	36.15 ± 2.56
	Arbuscule abundance in root system [A%]	30.75 ± 2.83	27.51 ± 0.99
Trap roots	Small trap roots (< 150 µm) containing storage organs [%]	0.23 ± 0.20	1.25 ± 0.66
	Large trap roots (> 150 µm) containing storage organs [%]	6.74 ± 1.02	7.43 ± 4.16
	Storage organs in colonized small trap roots [n per root]	2.29 ± 0.66	1.75 ± 0.88
	Storage organs in colonized large trap roots [n per root]	7.76 ± 3.00	4.38 ± 2.19
Soil core	Hyphae dry matter [mg per 250 ml soil]	n.d	14.7 ± 3.37
	Hyphal length [cm cm^−3^ soil]	2.88 ± 0.26	79.56 ± 4.48
	Storage organ abundance [n cm^−3^ soil]	67.25 ± 11.0	101.58 ± 34.9

In contrast, we could wash off many fewer hyphae from the soil compartments containing loam than from those containing sand (Table 1). In sand, the hyphal length density was one order of magnitude larger (approx. 80 cm cm^−3^) than in loam (approx. 3 cm cm^−3^). However, the mean soil spore abundance appeared somewhat similar in sand and loam.

The intense mycorrhizal colonization effectively contributed to plant growth and plant nutrition in both loam and sand (Table S1). In loam and sand, inoculation led to a substantial growth increment of approx. 9 and 10 g dry weight, respectively, compared to the non-mycorrhizal treatment. This accounted for an eightfold plant growth increment attributable to mycorrhizas in both soils. The biomass of AM and NM plants were very similar in the two soils. While N was significantly more concentrated in leaves of NM plants compared to AM plants in both soils, the concentrations of leaf P remained unaffected by inoculation and was generally low. This resulted in an average leaf N/P ratio of 43 in NM plants and 31 in AM plants in loam. In sand, the average leaf N/P ratio was 40 in NM plants and 23 in AM plants. These high N/P ratios verify relative P limitation of plant growth and that mycorrhizas alleviated the P limitation. That the leaf P concentration stayed approximately constant across inoculation treatments, but AM plants grew larger than NM plants, indicated that P was the growth limiting mineral nutrient overall.

### Pot water dynamics

AM plants transpired more water than NM plants which were smaller than AM plants. During the final twenty days of each experiment, an average of 50.8 ± 1.5 ml d^−1^ in NM pots and 87.8 ± 1.4 ml d^−1^ in AM pots (*N* = 4, ± se, P < 0.001) in loam were required to compensate daily water losses. In sand, 20.6 ± 0.5 ml d^−1^ in NM pots and 38.4 ± 2.6 ml d^−1^ in AM pots (*N* = 4, ± se, P = 0.004) were required. Obviously, the increases of evapotranspiration because of mycorrhizas were much lower (< twofold) than the increases of plant biomass. Therefore, evaporation from the pot surface dominated evapotranspiration. To illustrate this, we took the daily irrigation requirements at the beginning of the experiment as a proxy for evaporation, when transpiration of plants in the two-leaf state was negligible. In the case of sand, the daily irrigation requirement across the 3 initial days was 17.5 ml d^−1^ and equal between treatments. Assuming this evaporation rate to be valid also towards the end of the experiment, this would result in a mean transpiration rate of approx. 3 ml d^−1^ in NM and 21 ml d^−1^ in AM pots with sand. This accounts for a sevenfold increase of water use. This better matches the size relation of plants (eightfold higher biomass of AM plants) but introduced only a small bias to the pot water dynamics (Table 2). The AM inoculation of both soils led to a significant reduction of the median volumetric water content and the minimum water content in pots during the whole experiment. The higher evapotranspiration rates in loam than in sand were attributable to the climatic conditions (see Fig. S2), and therefore, the median water content in loam deviated more from the target water contents (20% and 12% in loam and sand, respectively) than in sand. Transpiration by AM plants reduced the median water pot water content by 1.6% in loam and by only 0.2% in sand. This also was a consequence of the low evapotranspiration rates in sand. It would have required a more frequent irrigation regime in loam than we employed to most effectively compensate the high transpiration of AM plants.

**Table 2:** Volumetric water contents observed in pots during culture of 10 to 11-week old maize plants inoculated (AM) or not (NM) with *R. irregularis*. The values show the median and minimum (± se) water contents of four biological replicates each for maize cultivated in loam soil and in quartz sand and were compared by *t* test. The volumetric water contents were calculated by subtracting the weights of all non-soil parts and the soil dry weight from the total weight of pots to get the gravimetric water content. The gravimetric water content has been recalculated to volumetric water contents with the bulk density in pots. The plant bias is calculated as the water content in the gravimetric method that is overestimated by the final shoot fresh weight and was considered to be valid for half of the growing period

	Volumetric water contents in pots [%]			
	Target	Median [± se]	Min [± se]	Plant bias [%]
**Loam (35% sand)**				
AM	20	12.2 ± 0.09	7.18 ± 0.14	0.05 ± 0.02
NM	20	13.8 ± 0.11	9.87 ± 0.10	1.00 ± 0.07
* P*		**< 0.001**	**< 0.001**	**< 0.001**
**Quartz sand (100% sand)**				
AM	12	11.3 ± 0.05	6.6 ± 0.30	0.15 ± 0.02
NM	12	11.5 ± 0.05	8.6 ± 0.06	1.34 ± 0.11
* P*		**0.009**	**< 0.001**	**< 0.001**

### Water retention in root-free soils

**Fig. 1:**
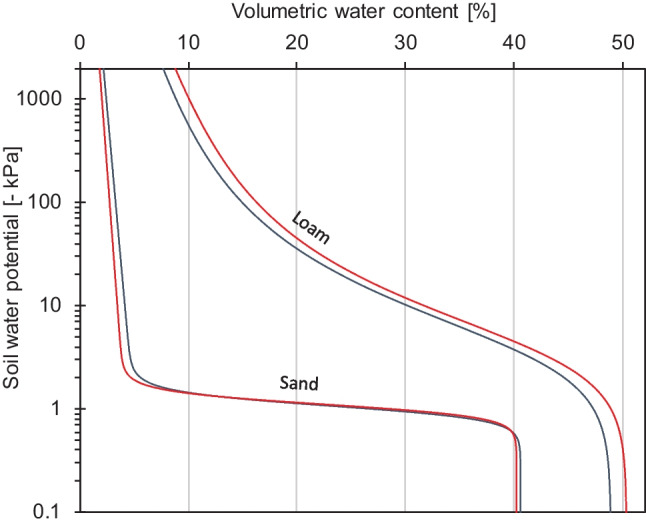
Water retention curves of root-free loam and quartz sand are shown for soils that either contained biomass of *R. irregularis* (blue lines) or not (red lines). Each line represents a fit of the Peters-Iden-Durner variant of the van Genuchten model to data obtained from 4 biological replicates

Within the root-free hyphal compartments, the water retention (indicated by Θ in Table 3) of loam decreased when AMF were present, while it increased in sand (Table 3 and Fig. 1). The curve shapes can be considered significantly different because some of the curve shape parameters showed non-overlapping confidence intervals in both soils (Table 3). Although the differences between AM and NM soils appear to be small in Fig. 1, note that the soil water potential is displayed on a log scale.

**Table 3:** Estimated curve shape parameters of the coupled unimodal constrained van Genuchten water retention and Mualem hydraulic conductivity model are given for treatment-wise fits to measurement data of four biological replicates of soil volumes that either contained *R. irregularis* (AM) or not (NM). The numbers show the estimated parameters with 95% confidence intervals in parenthesis for loam and sand. Non-overlapping confidence bounds between treatments within each soil type are highlighted in bold, and the root mean squared error is given for the water retention (RMSE Θ) and the hydraulic conductivity (RMSE K) function. For model theory and equations, please see Pertassek et al. 2015)

	Loam		Sand	
Model parameter	AM	NM	AM	NM
α [cm^−1^]	0.0286 (0.0274–0.0298)	0.0275 (0.0265–0.0284)	**0.0959 (0.0948–0.0971)**	**0.0923 (0.0912–0.0934)**
n [-]	1.614 (1.568–1.664)	1.656 (1.618–1.696)	6.654 (6.249–7.090)	7.211 (6.743–7.717)
Θ_r_ [cm^3^ cm^−3^]	**0.146 (0.134–0.158)**	**0.172 (0.164–0.180)**	**0.049 (0.047–0.052)**	**0.041 (0.039–0.043)**
Θ_Sat_ [cm^3^ cm^−3^]	**0.489 (0.488–0.490)**	**0.503 (0.502–0.504)**	0.405 (0.404–0.407)	0.402 (0.399–0.405)
K_Sat_ [cm d^−1^]	2384 (1044–5447)	1216 (629–2350)	**0.424 (0.384–0.468)**	**0.770 (0.600–0.990)**
τ [-]	1.365 (0.823–1.907)	0.860 (0.459–1.261)	−1 (−1 to −0.794)	−1 (−1 to −0.807)
ω	6.59 (2.91–15.0) × 10^−6^	14.5 (7.78–26.9) × 10^−6^	**15.6 (13.0–18.7) × 10**^**−3**^	**7.89 (5.95–10.5) × 10**^**−3**^
RMSE Θ	0.0091	0.0082	0.0201	0.0300
RMSE K	0.0547	0.0738	0.4814	0.4969

To further illustrate the magnitude of influence the mycorrhizal fungus had on the soil water potential, we fitted the hydraulic model to the data of the individual replicates and interpolated the soil water potential at reference levels of volumetric water content. The evaluation revealed that the soil water potential is most affected by the mycorrhizal fungus in the dry regime (Table 4) in both soils. While AMF increased the soil water potential by as much as 771 kPa at a volumetric water content of 10% in loam, AMF decreased it by 253 kPa in sand in comparison to NM soils at 3% water content. While the water potential in the more flexible loam soil is affected by the mycorrhizal fungus over the whole moisture range, these biotic influences are restricted to the drier portion of the range in quartz sand (Table 4). Vice versa, lower soil water contents in AM loam than NM loam corresponded to the same soil water potential (Table S2). The opposite was the case in sand (Table S2).

**Table 4:** Soil water potential (Ψ) interpolated at reference levels of volumetric water contents (Θ) are shown for root-free loam and sand volumes that were either populated by *R. irregularis* (AM) or not (NM). The values are the mean (± se) of four biological replicates and were compared by *t* test on log transformed data. *P* values lower than 0.05 are indicated in bold

		Ψ [kPa] at Θ = 40%	Ψ [kPa] at Θ = 30%	Ψ [kPa] at Θ = 20%	Ψ [kPa] at Θ = 10%	Ψ [kPa] at Θ = 5%	Ψ [kPa] at Θ = 3%
Loam	AM	−3.67 ± 0.10	−9.99 ± 0.21	−36.35 ± 2.43	−625.1 ± 107.8	-	-
	NM	−4.42 ± 0.08	−11.63 ± 0.31	−46.54 ± 3.18	−1396.8 ± 180.4	-	-
	***P***	**< 0.001**	**< 0.001**	**0.022**	**0.005**		
Sand	AM	-	−0.95 ± 0.03	−1.11 ± 0.02	−1.37 ± 0.05	−3.85 ± 1.72	−337.6 ± 147.9
	NM	-	−0.98 ± 0.04	−1.13 ± 0.04	−1.34 ± 0.05	−1.78 ± 0.07	−84.2 ± 39.9
	***P***		0.302	0.374	0.376	0.137	**0.031**

A close look at the data reveals that in loam the water potential under high water contents increased because of presence of the mycorrhizal fungus (Fig. S6). At low water contents, the elevated water potentials in hyphal compartments with the fungus likely were in consequence of greater drying in AM than NM pots at harvest. In sand, the water potential at high water contents remained unaffected by the presence of the mycorrhizal fungus but decreased at low water contents (Fig. S6). In contrast to loam, the soil water potential in sand was independent of the degree of drying of pots at harvest at 3% volumetric water content (Fig. S6).

**Fig. 2:**
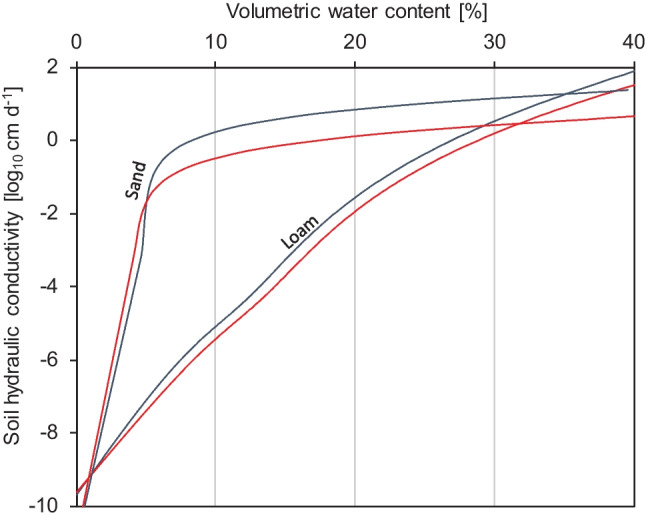
Unsaturated hydraulic conductivity of root-free loam and sand shown as a function of volumetric water content for soils that either contained biomass of *R. irregularis* (blue lines) or not (red lines). Each line represents a fit of the Mualem model to data from 4 biological replicates (soil cores)

Related to the changes in soil water potential, we observed that soil hydraulic conductivity was higher in AM than NM loam. Vice versa, in sand in the dry range, hydraulic conductivity from NM pots was higher than in sand from AM pots (Fig. 2). Again, note that that hydraulic conductivity is displayed on a log-scale.

**Fig. 3:**
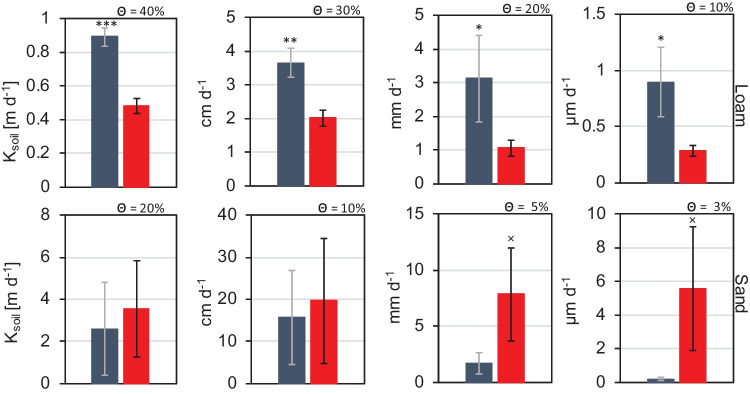
Unsaturated hydraulic conductivity (K_Soil_) interpolated at reference levels of volumetric water contents (Θ) are shown for root-free loam (top) and sand (bottom) volumes that were either populated by *R. irregularis* (AM, blue bars) or not (NM, red bars). The values are the mean (± se) of four biological replicates (*t* test; ***, *P* < 0.001; **, *P* < 0.01; *, *P* < 0.05; × , *P* < 0.1)

While the hydraulic model gave good treatment-wise fits in loam, the RMSE for sand was not satisfactorily small for hydraulic conductivity (see RMSE in Table 3). Especially, extrapolation from the model under high volumetric water contents should be interpreted cautiously (see Fig. S4). Therefore, we interpolated hydraulic conductivity values for the reference volumetric water contents from the raw measurement data for every individual soil sample. In doing so, we observed a significant increase in soil hydraulic conductivity in AM loam, which was up to threefold higher at low volumetric water contents than in NM loam (Fig. 3). In sand, the opposite, i.e., a reduction in hydraulic conductivity in sand from AM pots at low water contents, was observed. Because of the steep decline of hydraulic conductivity with low water content in the dry end in sand, there was a high variance in hydraulic conductivity. Therefore, we found only marginal significance (*P* < 0.1) between treatments in sand, but the opposite trend than we observed in loam.

For both investigated soils, the relationship between the soil water potential and hydraulic conductivity remained unaffected by the inoculation treatment (see Fig. S7).

### Water depletion in hyphal compartments

Because we expected differences in pot water dynamics when AM plants grow better than NM plants, we tested whether mesh would impose a limitation to water equilibration between the hyphal compartments and the surrounding soils. Indeed, this was found (Figs. S8, S9). At the median soil water contents of 12.2% and 13.8% in loam (see Table 2), the introduction of the nylon mesh substantially increased the water potential difference between the soil volume underneath and that above the mesh compared to when mesh was absent (Fig. S8). Because water potential gradients arise when conductivity is low, Fig. S8 illustrates that the mesh reduced hydraulic conductivity between the two loam soil volumes. This was not observed in sand at the median water contents of 11.5 and 11.3% (Fig. S8). Hence, we need to consider that in loam, water equilibration between the pot soil and the hyphal compartment was restricted by the mesh, while in sand, water could move quite freely between the soil in the main pot and the soil in the hyphal compartment.

**Fig. 4:**
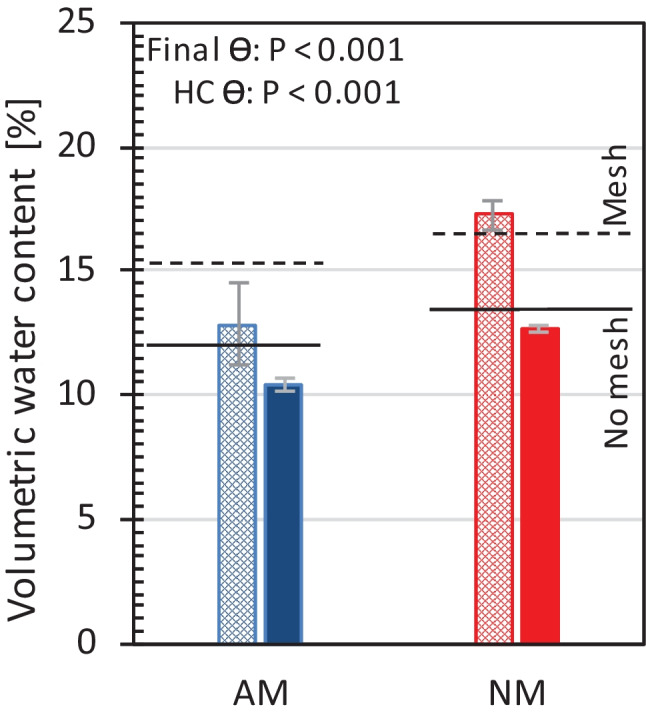
Volumetric water content (Θ) in hyphal compartments (HC, hatched bars) with loam after 11 weeks of maize growth is shown and compared to the final water content in pots after 1 day of withholding water (closed bars). The mean (± sd) of four replicates is shown for pots inoculated with *R. irregularis* (AM, blue) and their non-mycorrhizal counterparts (NM, red). The final volumetric water content in pots was corrected for the shoot fresh weight at harvest. The differences between inoculation treatments were compared by *t* test for independent samples and the *P* values are shown. The solid black lines indicate the expected volumetric water content in hyphal compartments 2.5 cm distant from a soil volume not separated by a mesh. The dashed black lines indicate the expected water content in hyphal compartments for the condition in which a 20-µm nylon mesh is in place at half the distance. These estimates were derived from interpolating the expected volumetric water content from the water retention curve at the soil water potentials that were measured either at the lower part of the soil volume or beneath a mesh (see solid lines in Fig. S8)

As the mesh seemed to affect water exchange in loam, we determined the volumetric water content in the hyphal compartments with loam finally without another irrigation dose at the day of harvest (Fig. 4) and compared this to the theoretical scenarios in which either a mesh is in place or not (black dashed and solid reference lines, respectively, in Fig. 4). Indeed, the mesh reduced water exchange between the pot soil and the hyphal compartments in loam. In both NM and AM pots, the final volumetric water content of the pot soil was lower than the final water content inside the hyphal compartments. But the mycorrhizal fungus seemed to counteract nylon mesh inhibition of water exchange. The volumetric water content of loam in hyphal compartments from NM pots was more than 4% higher (17.2%) than in the main pots at harvest (12.7%). In AM pots, the water content in the hyphal compartment (12.8%) was more similar to the water content in the main pots (10.4%) at harvest. In addition, the water content in the hyphal compartments from AM pots was not different from the expected water content in the inner compartment soil when no mesh separates the two soil volumes (solid black reference line in Fig. 4). But the water content in hyphal compartments of AM pots was reduced more strongly than expected for the condition in which a mesh separates the soil volumes (dashed black line in Fig. 4). The opposite is the case for NM pots. In the fungus-free scenario, the water content in the hyphal compartment at harvest did not differ from the expected water content when the inner compartment soil would be separated by a mesh in loam.

## Discussion

In this study, we hypothesized that mycorrhizal fungus ingrowth into the soil pore space increases soil water retention and decreases hydraulic conductivity in the fine-textured loam and causes the opposite in the coarse-textured sand. Our hypothesis is confirmed, especially for the driest soils. Therefore, our findings have implications for physiological plant water stress responses and for how drought responses of mycorrhizal plants should be interpreted.

To investigate our hypothesis, we aimed at producing comparable abundances of the mycorrhizal fungus in the two soils with contrasting textures. We achieved this by applying soil specific irrigation, fertilization, and timing of the harvest. The AM plants were similar in size; intensely mycorrhizal and the mycorrhizal fungus had covered the same distance within the pot soil in both experiments. We demonstrated that intense root colonization and a positive growth response mainly depended on the plant internal P limitation, regardless of the soil texture. Despite the substantially different physico-chemical properties of loam and sand, similar plant growth and root colonization rates were achieved in both soils when the added P was matched with the P fixing capacities of the soils. That tissue P concentrations of mycorrhizal plants did not differ from the plant P concentrations of the much smaller NM plants verified that plant P availability was the main constraint to plant growth. Furthermore, the high plant N/P ratios suggest that plants suffered from strong relative P limitation in both soils. These similarities in plant sizes, mycorrhizal growth responses, P limitations, and root colonization rates across loam and sand indicate that the mycorrhizal fungus had comparable prerequisites to colonization of the hyphal compartments.

Moreover, the intentionally induced P limitation kept all plants (irrespective of their growth response to mycorrhizas) small in relation to pot size. Importantly, this allowed us to avoid extreme differences in pot water dynamics across NM and AM treatments because the main water loss from pots was caused by evaporation. We did not fully decouple pot water dynamics from hyphal compartments with an air gap because (i) the air gap can impede hyphal proliferation (Khalvati et al. 2005) and (ii) the wet-dry dynamics in soils specific to AMF may indeed be a relevant ecological trait that influences soil physical properties (Hallett et al. 2009).

Despite intense root colonization rates in both loam and sand, we found much lower hyphal length densities in the hyphal compartments than others have found. For example, Joner and Jakobsen (1995) found several m of AMF hyphae per cm^3^ of soil. Besides the possibility that soil abundances of hyphae were truly lower in our case than in other studies, we also must consider that the hyphae were detached from plants at harvest and the hyphal compartments were subjected to hydraulic property measurement before hyphae were extracted. The water retention measurements in the lab lasted 12 to 15 days per sample. AMF hyphae can turn over rapidly in about 5 to 6 days (Staddon et al. 2003). Therefore, our lab procedure may have reduced the hyphae that can be extracted from soils. Such reductions in hyphal abundance may influence soil hydraulic properties (Querejeta et al. 2012), which we cannot exclude here. Indeed, because the abundance of spores which are persistent in soils matched with the numbers that others have found for *R. irregularis* (Jansa et al. 2002) or in maize cropping systems (Bhadalung et al. 2005), degradation of the hyphae might have taken place during the water retention measurements. That we could extract more hyphae from sand than from loam may reflect hyphae being somewhat stable against degradation in sands. Drew et al. (2003) showed that AMF hyphae are thicker in sand than in finer textured soils, which may suggest a higher stability of hyphae in sands. Fine hyphae also may be hard to extract from loam because of the large charged surface area of the clay component to which hyphae can bind. Although we only can speculate about true abundances in the hyphal compartments of our experiments, we clearly verified that the mycorrhizal fungus did populate the compartments and that the preconditions for production of extraradical hyphae were widely comparable in loam and sand. Moreover, apart from intact hyphae, fungal exudates and debris also may explain our findings because organic matter additions to soils effectively modulate their hydraulic properties (Rawls et al. 2003).

### Possible factors for the observed changes in soil water retention

We observed a decrease of water retention in AM loam. Our finding is consistent with observations of Augé et al. (2001) in a loamy potting mix with cowpea and *R. irregularis*, with Bitterlich et al. (2018b) in a vermiculite-containing substrate with tomato and *Funneliformis mosseae* and with Neergaard Bearden (2001) in a fine-textured vertisol with sorghum colonized by several AMF species. The common feature of all studies is the use of substantial amounts of flexible, low weight substrate components that swell and shrink and can be aligned by organisms. The differences to our study are that the investigated soil samples contained plant roots (Augé et al. 2001; Bitterlich et al. 2018b) and that water retention was measured merely on extracted soil aggregates (Neergaard Bearden 2001). We show for the first time that soil water retention also responds to AMF ingrowth in otherwise undisturbed, root-free soils.

Our observed increase of water retention in AM sand is in agreement with a study using a substrate made of coarse spoil (Daynes et al. 2013) and with another that used a potting mix dominated by rigid materials (coarse sand and zeolite) (Pauwels et al. 2020). Again, in both studies, the investigated potting mixes also contained roots. Pauwels et al. (2020), however, also included the same root exclusion compartments as in this study and consistently found increased water retention when *R. irregularis* colonized the rigid substrate in the hyphal compartment. The observed responses of water retention in both soils correspond to effects of soil organic matter additions and fit with concepts of soil structure.

Multiple abiotic and biotic factors interdependently determine the water retention of a soil with a specific texture (Bronick and Lal 2005; Rawls et al. 2003). The factors involve soil bulk density, organic matter, and wet-dry dynamics. In our experiment, changes to the soil bulk density were diminished by the restriction that the mesh imposed to particle movement. Furthermore, the few mg of mycorrhizal fungus dry matter added to 300 g dry loam and 387 g dry sand in hyphal compartments were negligible for soil bulk densities. Nevertheless, both loam and sand were initially poor in organic matter, the condition under which soil water retention responds most sensitively to organic matter additions (Rawls et al. 2003). Therefore, the newly introduced organic matter of mycorrhizal fungus origin into both soils qualifies as a possible mechanism for the observed changes in water retention in this study.

The decrease of water retention in loam and its increase in sand upon mycorrhizal fungus ingrowth corresponds with expectations for organic matter additions to both soils with low initial quantities of organic matter (Rawls et al. 2003). In contrast, increasing water retention in both loam and sand would be expected upon further additions of organic matter to soils initially rich in organic matter (Rawls et al. 2003). Hence, we likely observed antithetic responses of water retention to AMF ingrowth in loam and sand because AMF added organic matter to soils originally containing less than 1%. Additionally, we contend that most added organic matter in the hyphal compartments is of mycorrhizal fungus origin because plant exudates are not expected to “travel” much further than the length of root hairs through nylon meshes (Sauer et al. 2006), while hyphae grow many centimeters away from roots.

The antithetic response of water retention to mycorrhizal fungus ingrowth in loam and sand likely relates to the specific susceptibility to aggregation of their mineral particles. For loam, we can expect that aggregation took place even without mycorrhizal fungus ingrowth because the loam contained considerable amounts of (light) clay and CaCO_3_ (as indicated by the pH) as a cementing agent. Sands with large particles are resistant to aggregation because they lack a small mineral fraction and cannot be compacted (Nimmo 2004a).

Hyphal organic matter likely adds organic binding agents to soils with texture dependent consequences. In soils such as loams which are susceptible to aggregation, AMF can stabilize aggregates against disintegration and can bind existing small aggregates into large ones (Rillig and Mummey 2006). In contrast, in sand, newly introduced organic matter intercalates within the inflexible sand matrix, which reduces the diameter of the large pores, blocks pore necks, and covers the relatively small surfaces of the solid soil phase. In AM loams, both the reduced saturated water content and the lower water retention in the wet range are consistent with increased aggregation that resisted the saturation procedure of our samples. The 1.4% decrease of saturated water content in AM loam more likely reflects entrapped air (inside aggregates) than the fungus volume. Considering conservative size relations of extraradical *R. irregularis* structures (spherical spores with _~_ 150 µm diameter and cylindrical hyphae with _~_ 10 µm diameter) and the quantities found, the mycorrhizal fungus would have added only 0.0001 cm^3^ of fungal biomass to 1 cm^3^ of soil, i.e., 0.01% volumetric water content. Hence, a large proportion of entrapped air the in the saturated mycorrhizal loam would increase soil hydrophobicity (Hallett 2008) and reduce water access in pores. Increased water repellency has been documented for soils that were newly populated by AMF hyphae (Rillig et al. 2010) which protect aggregates against infiltration and, hence, reduce disruption during re-wetting.

The effects of entrapped air on the soil water retention curve would quickly vanish under subsequent drying (Stonestrom and Rubin 1989), but in the wet range of the water retention curve, the stability of soil aggregates would contribute increased water release. The soil water potential should increase at high soil water contents because of an elevated abundance of large pores outside aggregates that drain until reaching −10 kPa of soil water potential (Guber et al. 2003). Our observations for AM loams are consistent with that. Another possibility is that mycorrhizal fungus organic matter, similar to plant exudates (Naveed et al. 2019), decreases the surface tension of soil water which increases drainage from pores.

In the dry range, the soil water potential of loam **responded** to the degree of drying across AM and NM pots. At low water potentials, water is expected to be released from (small) pores inside aggregates and depends on the surface properties of the soil particles (Guber et al. 2003). That the water potential at low water contents in AM loam increased with the drying may suggest an increased disintegration of large aggregates (Jastrow et al. 1998). Large aggregates that formed in AM loam may not have been stable enough to withstand the higher tensile forces that developed in rapidly drying AM loam.

In sand, the possible aggregation-driven effects on water retention should be absent (Nimmo 2004a). Consistently in sand, mycorrhizal fungus ingrowth caused neither changes of saturated water content nor changes of water potential in the wet range. The possible reason for these observations is that the likelihood for persistent entrapped air after saturation in sand is low because stable aggregates could not form. Moreover, even if aggregates did form in sand after mycorrhizal fungus ingrowth, water retention would be unlikely to decline (as in loam) because in any case, almost the whole pore space in sand is subject to draining. Instead, the observed increase of water retention in mycorrhizal sands agrees with how plant-derived mucilage influences soil water retention in coarse sand (Kroener et al. 2018). Sticky and viscous mucilage connects particles and stays hydrated when relatively moist. When the soil dries, the viscous mucilage shrinks but sustains the contact at the organo-mineral surface, which diminishes matric potential and increases water retention (Kroener et al. 2018). Mucilage effects cease under extremely dry conditions close to the permanent wilting point at −1500 kPa (Naveed et al. 2019). Notably, AMF and their organic matter are considered to be sticky (Miller and Jastrow 2000), and our observation of the water retention by the AM sand is in agreement with the mechanisms attributable to mucilage. In summary, mycorrhizal fungus ingrowth caused opposite responses of water retention in loam and sand, which furthermore manifested in modulated soil hydraulic conductivities.

### Possible factors for the observed changes in hydraulic conductivity in soils and across nylon meshes

In the past, reduced path lengths for water in soils with AMF often have been discussed/inferred as a mechanism that facilitates root water access, supposedly mediated by hyphae bridging air-filled pores in the soil or at the root-soil interface. This suggests elevated hydraulic conductivity in soils with AMF because hydraulic conductivity depends on the tortuosity of the water path. However, we know of only one study that measured hydraulic conductivity in a particular root-free substrate with AMF (Bitterlich et al. 2018a). Their findings are consistent with our observations of loam.

Although the degree of tortuosity in soils determines how fast a unit water volume can move from one place with high water potential to another with lower water potential, our findings do not indicate that soils containing the mycorrhizal fungus possessed reduced water path lengths. In both tested soils, the relation between hydraulic conductivity and soil water potential remained unaffected by AMF ingrowth (Fig. S7). Hence, the changes to hydraulic conductivity we observed in AM loam and sand at certain soil water contents were a direct consequence of the affected soil water potential. Therefore, we infer that the presence of the mycorrhizal fungus alters pore size distribution, possibly via aggregation in loam and via pore clogging (reduction in pore diameter) in sand. The mycorrhizal fungus caused a relative gain in large (inter-aggregate) pores in loams and a relative gain in small pores (due to diameter reductions of large pores) in sand. In consequence, a greater proportion of the soil water resides in easily drainable large pores with higher conductivity loams than in sands for which the opposite occurs. Nevertheless, we emphasize that the mycorrhizal fungus-induced influences on hydraulic conductivity are distinct from the effects AMF can have on water transport across root-exclusion meshes. We demonstrated that with our test on mesh introduction into the soils.

Indeed, it would require intact, bridging hyphae to transport water from a hyphal compartment to a plant if the compartment is isolated from the surrounding soil by an air gap or if the root-exclusion mesh itself constitutes a hydraulic disconnection as in our case in loam. In the tested loam, a strong water potential gradient between the two separated soil volumes developed during drying because mesh reduced hydraulic conductivity between the separated loam volumes. This happened although water retention of the loam was the same whether there was a mesh in place or not (see Fig. S9). The cause for this likely is that the loam shrank from the rigid mesh upon desiccation, thereby producing a gap. In contrast, because sand does not shrink water continues to move freely across the mesh. The substantial decrease in hydraulic conductivity per unit water potential with mesh in the loam illustrates the dependency of hydraulic conductivity on other factors than the water potential, i.e., on the tortuosity of the water path. The effective path length for water increased because of increasing loss of contact between the mesh and the loam during desiccation-induced shrinkage. In theory, an air gap causes infinite path lengths for (liquid) water out of hyphal compartments but path lengths become finite when hyphae bridge the gap. The higher water depletion rates in loam-filled hyphal compartments of AM than NM pots indicated higher a hydraulic conductivity across the mesh with hyphal bridges. This accords with studies that have shown that AMF facilitate water depletion from hyphal compartments that were hydraulically decoupled by air gaps (Kakouridis et al. 2022; Ruth et al. 2011). Indeed, AMF are highly capable of overcoming hydraulic insulation of a mesh.

In the case of an existing air gap regardless whether it is constructed intentionally, living hyphae may function as the most important pathway for water movement out of hyphal compartments. Bridging of an air-filled space, however, may not be the only relevant trait that modulated soil hydraulic conductivity inside continuous soil volumes. Instead, it seemed that the mycorrhizal fungus populating pore space altered the soil water potential at the same soil water content which resulted in different hydraulic conductivities. This is indicated by the fact that mesh introduction into loam reduced hydraulic conductivity per unit water potential, while mycorrhizal fungus ingrowth did not affect that relationship. Our findings may stimulate the discussion of whether the use of hydraulically isolated soil volumes only accessible to hyphae serve to investigate an ecologically relevant feature of AMF that facilitates plant water use. Such air gaps artificially force water accessibility from soils beyond the air gaps to depend upon hyphae cross sections. This implies two things: (i) whether water beyond air gaps can be depleted in detectable and ecologically relevant quantities would depend on plant access to water in the immediate vicinity of roots and (ii) any benefits for soil water uptake that AMF might provide that are not related to bridging hyphae (such as aggregation) are obviated because water movement is absolutely dependent upon gap-bridging intact hyphae. There is a chance that usage of nylon meshes tends to overestimate the contributions of intact hyphae to plant water uptake, especially when the separated soil volume is relatively large compared to the plant compartment. Nevertheless, there also is a chance that the use of mesh-separated hyphal compartments underestimates the contribution of AMF to plant water acquisition because the effects of hyphae on soil structure beyond the mesh are rendered irrelevant.

## Conclusions: the potential consequences of mycorrhizal fungi in soils for plant drought physiology

This study shows for the first time that water retention and hydraulic conductivity in root-free soils of different textures change when they are populated by a mycorrhizal fungus. This likely has consequences for the drought physiology of plants growing in soils with mycorrhizal fungi, probably even without a functional root association. Augé (2004) showed that non-host bean mutants that grew in a previously mycorrhizal soil maintained stomates open which suggests an alleviated water stress response. They used a loamy potting medium similar to the loam in the present study in which we found an increase in soil water potential across the plant-available moisture range when the mycorrhizal fungus was present. If our finding is general, a transient stimulation of stomatal conductance (Bitterlich et al. 2019) of any plant in such a soil with AMF could be expected, especially under low soil moisture because soil hydraulic properties rather than root hydraulic properties limit plant water uptake in dry soils. With an increased soil water potential (or soil hydraulic conductivity) for a given soil water content, plants would require fewer osmotic adjustments in roots to sustain the soil to root water flow, a condition that could be termed “less stressed.” In our case, we would expect the opposite for quartz sand. Mycorrhizal plants are expected to experience stronger water stress than NM plants in sands because the soil water potential and hydraulic conductivity of sand declined with mycorrhizal fungus presence. Studies that address plant water relations are difficult to carry out on pure sand, however, because it is difficult to manage pot water contents to precise soil water potentials under dry conditions. The shape of the water retention curve in sand (e.g., Fig. 1) illustrates these difficulties: it requires only a depletion of approx. 2% volumetric water content to go from wet conditions (−10 kPa) to lethal drought (−1500 kPa).

It is clear that soil water potential is a relevant drought stress parameter, and we showed that this changes in a texture specific manner when a mycorrhizal fungus occupies a soil. We emphasize that we found the greatest changes in soil water potential upon mycorrhizal fungus presence in ranges that lie beyond the measurement range of tensiometers which can be used in pots to control soil water tension. This bears the risk that commonly applied workarounds fail in conveying the same soil water potential to AM and NM plants. Using fractions of a priori determined soil water capacity values (as we did, too) to induce edaphic drought (or to maintain pots at a particular water content with theta probes) with AMF present could lead to elevated water potentials in fine textured soils and to reduced water potentials in coarse mineral potting media at the same soil moisture. Such (possibly unintentionally induced) discrepancies between inoculation treatments in soil water potentials may contribute to findings such as altered stomatal regulation and aquaporin expression by mycorrhizal plants under low soil moisture. We appreciate that this is a scenario which it is difficult to address experimentally. Nevertheless, this is potentially of high ecological importance and may contribute to the frequent findings of altered plant drought responses by mycorrhizal plants. Based on our findings, we recommend considering soil hydraulic properties as a variable in future studies on water relations of mycorrhizal plants. To fully understand the ecological relevance of AMF-induced modulations of soil hydraulic properties, future studies should investigate the response of soil hydraulic properties along gradients of hyphal densities and how the sensitivity of soil hydraulic properties to hyphal densities depends on soil texture and organic matter contents of soils.

## Supplementary Information

Below is the link to the electronic supplementary material.

Supplementary file1 (DOCX 1043 KB)

## Data Availability

The datasets generated during and/or analysed during the current study are available from the corresponding author on reasonable request.
