# Supplementary material for: An arbuscular mycorrhizal fungus alters soil water retention and hydraulic conductivity in a soil texture specific way
Source: Mycorrhiza. 2023 Mar 28;33(3):165–79. doi: 10.1007/s00572-023-01106-8 (PMC10244285; doi:10.1007/s00572-023-01106-8)
Supplement: Supplementary file 1 — Supplementary file1 (DOCX 1043 KB) [file 572_2023_1106_MOESM1_ESM.docx]

Fig. S1: A: Truncated conical 3 l pots (without drainage) filled with either sand or loam received two root-exclusion compartments. One is the hyphal compartment (grey cylinder) within the center of the pot, placed in a way that the core openings covered with 20 µm nylon mesh (blue hatched pattern) are standing upright. This way, mycorrhizal hyphae (violet thin lines) can spread laterally from downward growing roots (brown lines) into the hyphal compartment. The second compartment is a trap root compartment (hatched rectangle) next to the pot rim, covered with the same nylon mesh as the hyphal compartment. The purpose of the trap root compartment was to assess when the mycorrhizal fungus had grown the farthest from the plant shoot (green cylinder). B: The trap root compartments were biopsy pockets with four different sections (each 1 x 1 cm) filled with mats of dried maize roots. Hence, four chances were provided for verification of mycorrhizal colonization in trap roots. C: The horizontal alignment of the hyphal compartments guaranteed that ingrown hyphae were oriented in the direction in which water leaves the hyphal compartment when drawn horizontally from hyphal compartments by the transpiration stream. After removal, evaporation from the hyphal compartment soil during water retention measurements (the black cylinder is the measurement head for tensiometers inside the soil (texture filling)) occurs in a vertical orientation.


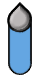


A

B

C


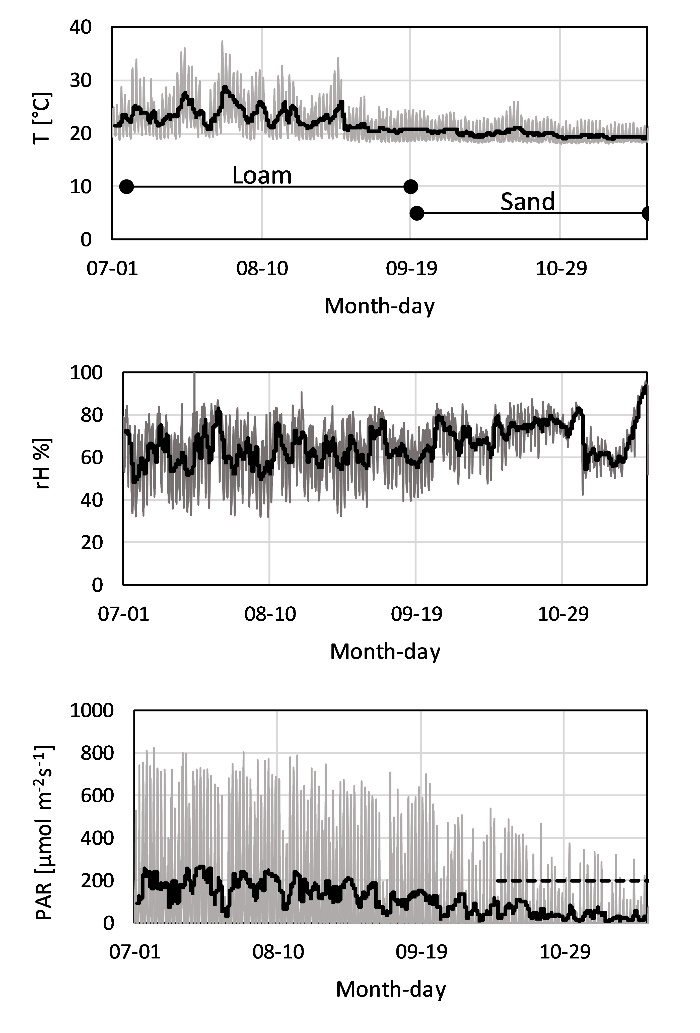
Fig. S2: Climatic conditions in the glass house over the duration of the two experiments carried out on loam and sand (indicated within the temperature plot, top). The grey lines show the actual condition logged at 12 min intervals, the black lines indicate the daily mean in temperature (top), relative humidity (middle) and photosynthetic active radiation (PAR, bottom). The dashed line in the plot for irradiance indicates the use of supplemental lighting which provided approx. 200 µmol m^-2^s^-1^ at canopy height for 8 hours during the photoperiod.


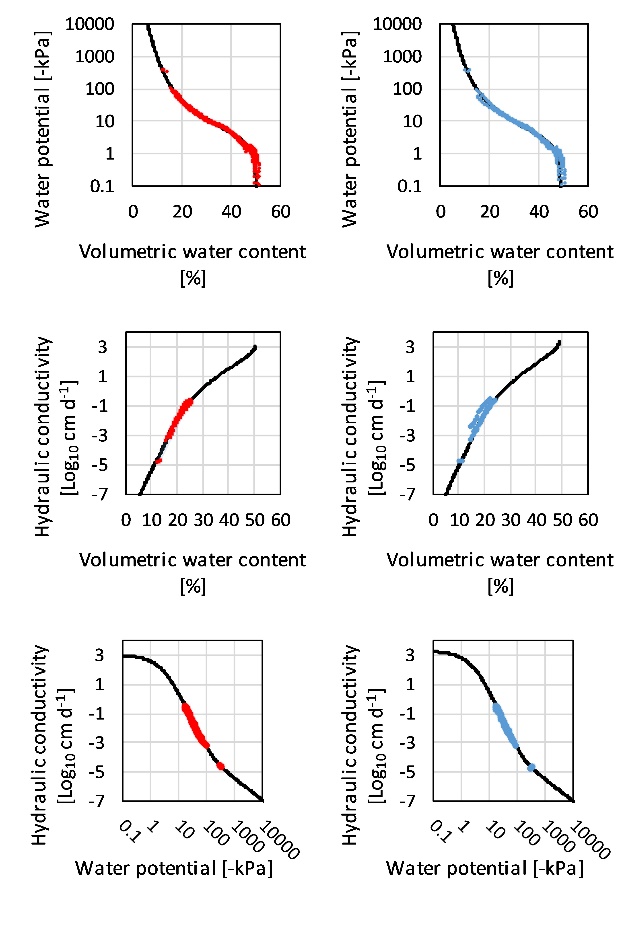
Fig. S3: Treatment-wise fits of the unimodal constrained van Genuchten model (black lines) to measurement data (colored dots) of four biological replicates in loam either without (red) or with *Rhizophagus irregularis* (blue).


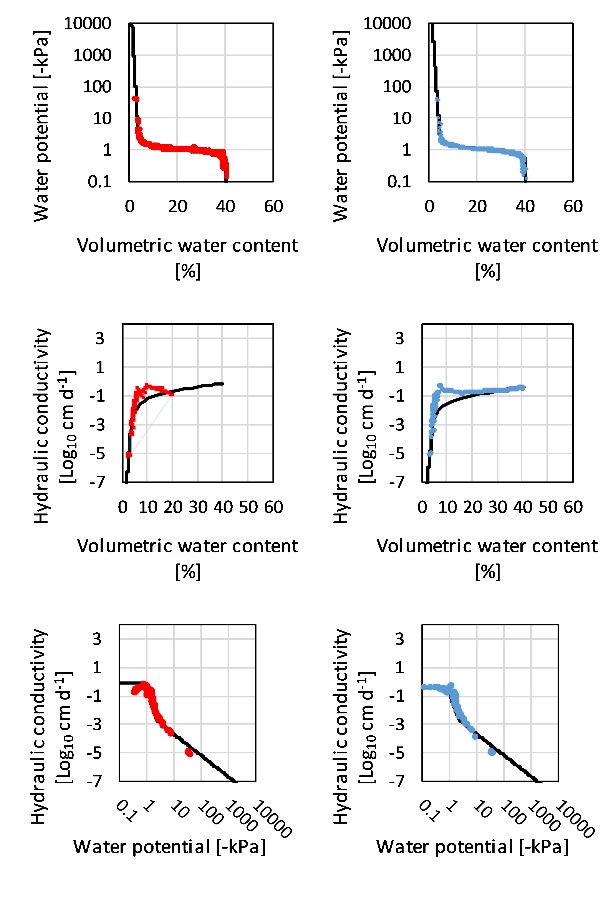
Fig. S4: Treatment-wise fits of the unimodal constrained van Genuchten model (black lines) to measurement data (colored dots) of four biological replicates in quartz sand either without (red) or with *Rhizophagus irregularis* (blue).


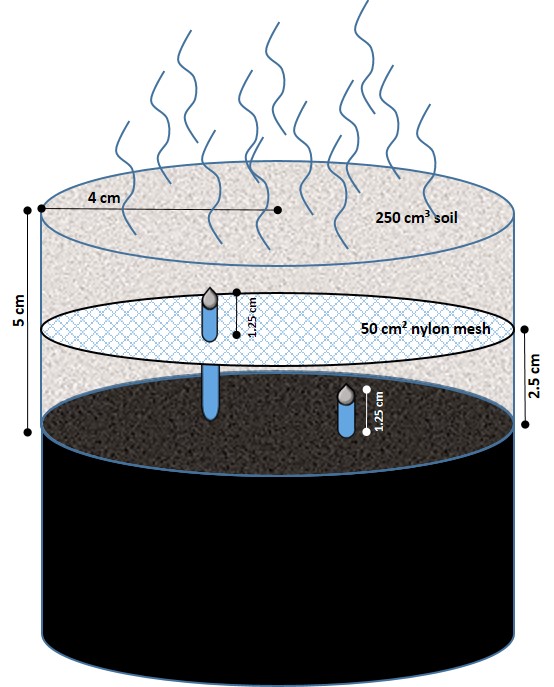
Fig. S5: Illustration of the setup for testing the flow impedance of a 20 µm nylon mesh introduced into soils. The two tensiometers filled with deionized water (blue) were located equidistantly above (top tensiometer) and below the mesh (bottom tensiometer) in a soil core filled with 250 cm³ soil (brown textured cylinder). The tensiometer tips (grey) were located at 1.25 cm (bottom) and 3.75 cm (top) height within the soil, while the mesh was installed equidistant between them at 2.5 cm soil height. During evaporation, water is drawn from the tensiometers through their tips into the desiccating soil and the hypopressure which develops inside the tensiometers is measured by the measurement head (black cylinder) until air entered both tensiometers and the tension dropped to 0 hPa. The hypopressure inside the tensiometers equals the water potential of the surrounding soil. The hydraulic conductivity is calculated from the water potential difference between the top and bottom tensiometers and the water loss per unit time.


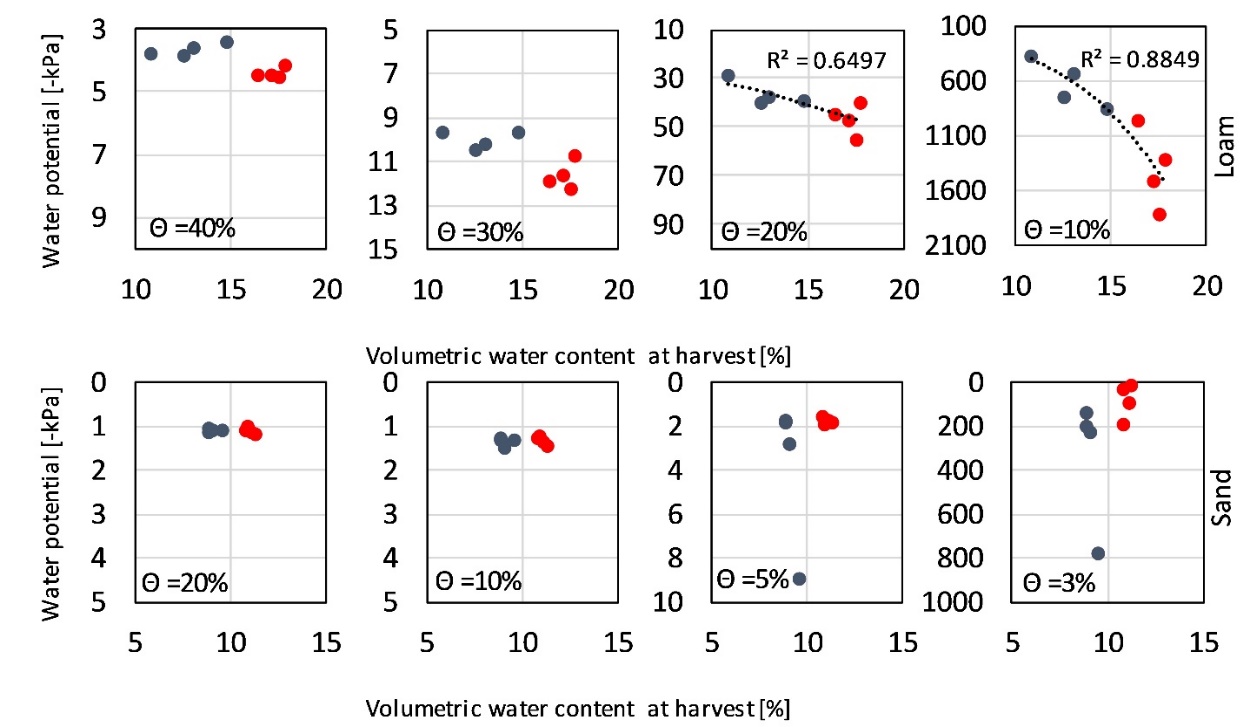
Figure S6: Soil water potential at reference volumetric water contents (Θ) of root-free loam (top) and quartz sand (bottom) is shown as a function of the volumetric water contents of the soils at harvest. The soils either contained biomass of *Rhizophagus irregularis* (AM, blue) or not (NM, red) in four replicates of each type. When a simple exponent regression (y = e^x^) across all data points was significant at α = 0.05, we included the dashed regression line and display the coefficient of determination (R^2^).

**
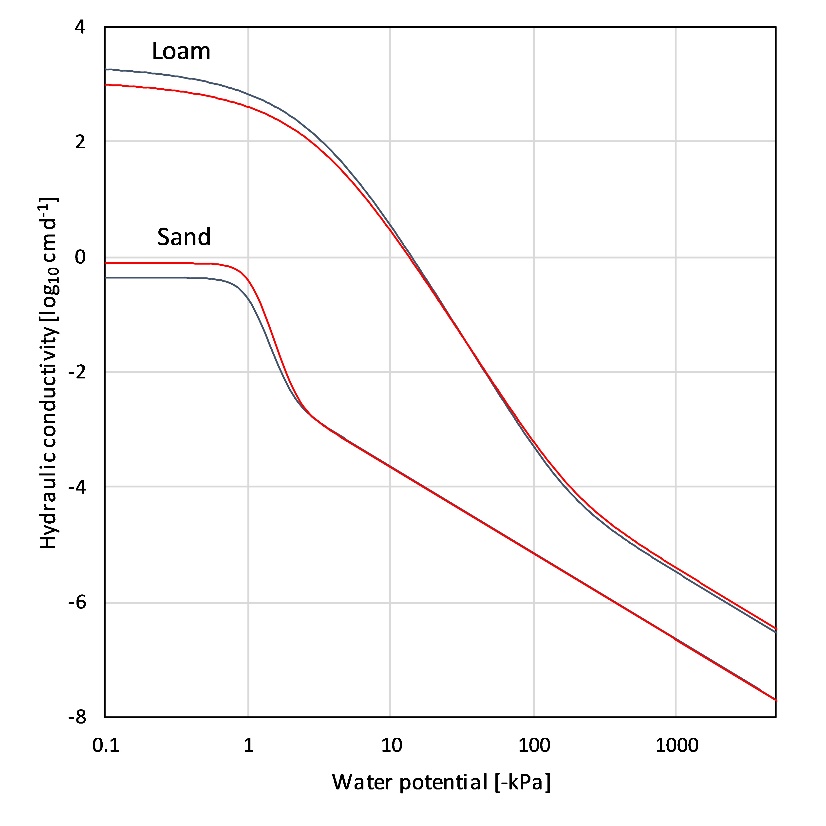
**

Fig. S7: Soil hydraulic conductivity of loam and sand as a function of the soil water potential for soils that either contained biomass of *Rhizophagus irregularis* (AM, blue lines) or not (NM, red lines). The lines represent treatment-wise model fits to data of four biological replicates. The data showed no significant differences between treatments.

**
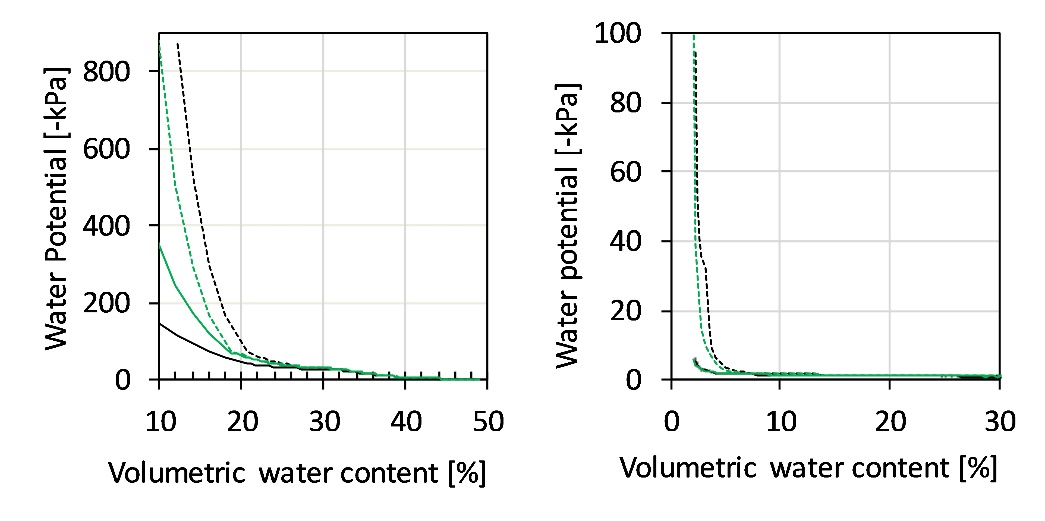
**

Figure S8: Soil water potential versus volumetric water content of a loam (left) and a quartz sand (right) in which two tensiometers were installed and which either contained a nylon mesh (20 µm opening, black lines) that separated the soil volumes horizontally or not (green lines). The dashed lines represent the tensiometer that was located above the height of the mesh (when present or not); the closed lines represent the tensiometer that was located beneath the height of the mesh. The lines represent mean values of three replicates.


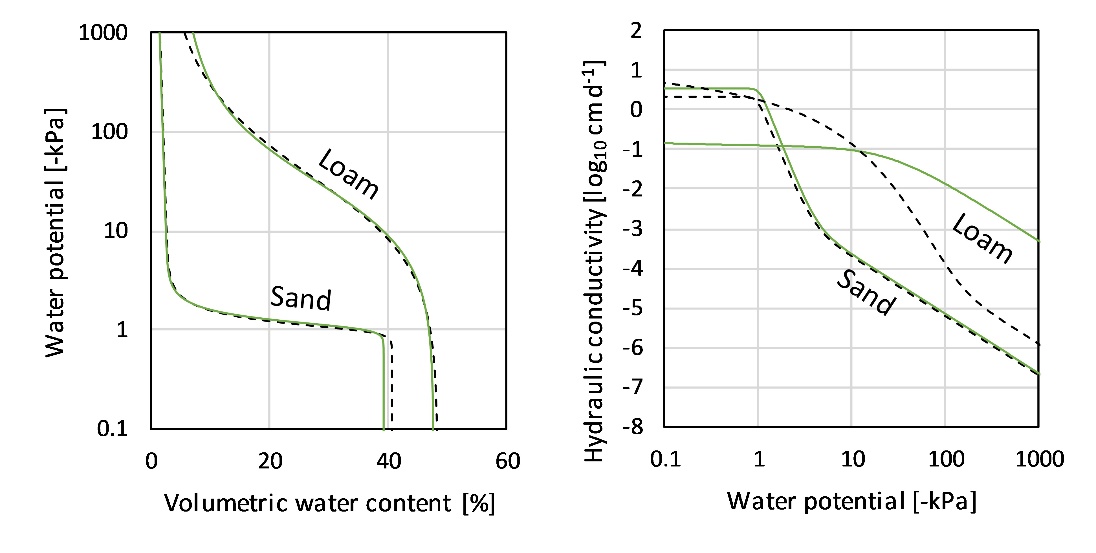
Fig. S9: Soil water retention (left) and hydraulic conductivity (right) versus the soil water potential of loam soil (Loam) and quartz sand (Sand) in which either a 20 µm mesh divided the soils (dashed black lines) or not (solid green lines). The lines each represent model fits of three replicates.

Table S1: Mean (± se) of four biological replicates for plant dry weight (DW), phosphorus and nitrogen concentrations in plant dry tissues of 10 to 11-week old maize inoculated (AM) or not (NM) with *Rhizophagus irregularis* in loam soil and in quartz sand. Within soil type, probabilities of difference by t-test are shown and those lower than 0.05 are in bold.

|  | Plant development | | | Plant nutrition | | | |
| --- | --- | --- | --- | --- | --- | --- | --- |
|  | Plant | Shoot | Root | Leaf P | Root P | Leaf N | Root N |
|  | [g DW] | [g DW] | [g DW] | [mg g^-1^ DW] | [mg g^-1^ DW] | [mg g^-1^ DW] | [mg g^-1^ DW] |
| **Loam (35% sand)** | | | | | |  |  |
| AM | 9.7 ± 0.94 | 7.2 ± 0.80 | 2.5 ± 0.65 | 0.63 ± 0.02 | 0.57 ± 0.03 | 19.4 ± 0.65 | 10.3 ± 1.10 |
| NM | 1.2 ± 0.19 | 0.8 ± 0.17 | 0.4 ± 0.06 | 0.68 ± 0.09 | 0.61 ± 0.06 | 29.3 ± 1.77 | 11.6 ± 0.73 |
| *P* | **<0.001** | **<0.001** | **0.019** | 0.662 | 0.552 | **0.002** | 0.343 |
| **Quartz sand (100% sand)** | | | | |  |  |  |
| AM | 11.5 ± 1.13 | 8.4 ± 0.87 | 3.1 ± 0.47 | 1.03 ± 0.12 | 0.55 ± 0.02 | 23.6 ± 1.50 | 14.6 ± 1.17 |
| NM | 1.4 ± 0.12 | 1.1 ± 0.11 | 0.3 ± 0.03 | 0.75 ± 0.03 | 0.53 ± 0.02 | 30.5 ± 1.50 | 19.3 ± 0.60 |
| *P* | **<0.001** | **<0.001** | **0.001** | 0.059 | 0.523 | **0.017** | **0.012** |

Table S2: Mean volumetric water contents (Θ) interpolated at reference levels of soil water potential (Ψ) of four biological replicates are shown for root-free loam and sand that either contained *Rhizophagus irregularis* (AM) or not (NM). Within soil type, probabilities of difference by t-test are shown and those lower than 0.05 are in bold.

|  |  | Θ [%] at  Ψ = -1 [kPa] | Θ [%] at  Ψ = -6 [kPa] | Θ [%] at  Ψ = -10 [kPa] | Θ [%] at  Ψ = -100 [kPa] | Θ [%] at  Ψ = -1000 [kPa] | Θ [%] at  Ψ = -1500 [kPa] |
| --- | --- | --- | --- | --- | --- | --- | --- |
| Loam | AM | 48.0 | 35.9 | 30.6 | 15.0 | 9.6 | 8.9 |
|  | NM | 49.2 | 37.9 | 31.8 | 16.5 | 10.9 | 10.1 |
|  | ***P*** | **0.048** | **< 0.001** | **0.009** | **0.019** | **0.013** | **0.012** |
|  |  |  |  |  |  |  |  |
| Sand | AM | 27.2 | 4.4 | 4.2 | 3.3 | 2.4 | 2.2 |
|  | NM | 29.9 | 3.8 | 3.5 | 2.5 | 1.8 | 1.7 |
|  | ***P*** | 0.261 | 0.055 | **0.045** | **0.024** | **0.021** | **0.022** |
|  |  |  |  |  |  |  |  |
